# Selective Light-Triggered Release of DNA from Gold Nanorods Switches Blood Clotting On and Off

**DOI:** 10.1371/journal.pone.0068511

**Published:** 2013-07-24

**Authors:** Helena de Puig, Anna Cifuentes Rius, Dorma Flemister, Salmaan H. Baxamusa, Kimberly Hamad-Schifferli

**Affiliations:** 1 Department of Mechanical Engineering, Massachusetts Institute of Technology, Cambridge, Massachusetts, United States of America; 2 Department of Biological Engineering, Massachusetts Institute of Technology, Cambridge, Massachusetts, United States of America; 3 Department of Biology, Massachusetts Institute of Technology, Cambridge, Massachusetts, United States of America; 4 Institut Químic de Sarrià, Universitat Ramon Llull, Barcelona, Spain; University of Helsinki, Finland

## Abstract

Blood clotting is a precise cascade engineered to form a clot with temporal and spatial control. Current control of blood clotting is achieved predominantly by anticoagulants and thus inherently one-sided. Here we use a pair of nanorods (NRs) to provide a two-way switch for the blood clotting cascade by utilizing their ability to selectively release species on their surface under two different laser excitations. We selectively trigger release of a thrombin binding aptamer from one nanorod, inhibiting blood clotting and resulting in increased clotting time. We then release the complementary DNA as an antidote from the other NR, reversing the effect of the aptamer and restoring blood clotting. Thus, the nanorod pair acts as an on/off switch. One challenge for nanobiotechnology is the bio-nano interface, where coronas of weakly adsorbed proteins can obscure biomolecular function. We exploit these adsorbed proteins to increase aptamer and antidote loading on the nanorods.

## Introduction

Nature has engineered the blood coagulation cascade to initiate clotting with temporal and spatial precision. However, our ability to artificially manipulate blood clotting is not nearly as precise. In wound healing and surgery, blood clotting must be controlled for patient safety, and is achieved predominantly *via* the anticoagulants heparin, warfarin and others. However, anticoagulants suffer major limitations, causing them to rank as the leading cause of death in adverse drug reactions in therapeutic use in the US [1], [2]. This is due to the fact that these anticoagulants can only inhibit the cascade, so their means of control is inherently one-sided. Furthermore, many anticoagulants suffer additional limitations. Heparin, the most prevalently used anticoagulant, is a polydisperse polymer that inhibits several species in the clotting cascade, but not a single specific factor. The main source of heparin is the intestines of livestock such as pigs, but the risk of contamination is significant and can be fatal [3]–[5]. More importantly, these anticoagulants have no specific antidote, and reversing their effect is predominantly achieved by clearance, which can vary greatly among people. Thus, introducing an inhibitor and a specific antidote with spatial and temporal control is highly desirable.

Nanotechnology has great potential as an enabling technology for biology because nanoparticles can be designed to interface directly with biomolecules. In particular, external laser excitation of nanoparticles can trigger payload release [6], [7], so nanoparticles can act as handles for controlling biological processes. Gold nanorods (NRs) have gained considerable interest for therapeutic applications because they can be selectively excited where tissue is transparent to release multiple species that can impact complex processes [6], [8]–[10] so they have many advantages for controlling blood clotting.

**Figure 1:**
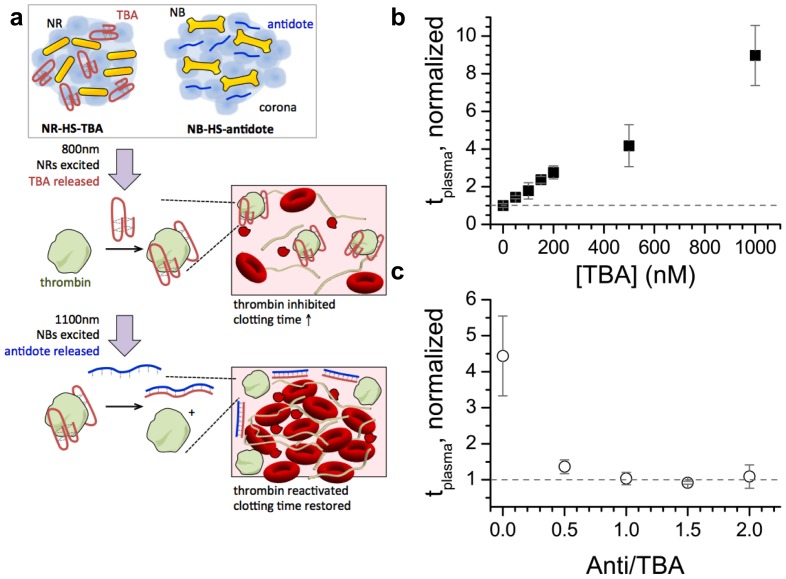
TBA and antidote affect coagulation in whole human blood. a) Schematic of coronas made from human serum (HS) loaded with NRs and TBA (NR-HS-TBA) + coronas loaded with NBs and antidote (NB-HS-antidote). 800 nm laser irradiation melts the NRs, triggering release of TBA from the coronas, which inhibits thrombin and causes blood coagulation times to increase. Following this, 1100 nm laser irradiation melts the NBs, triggering release of the DNA antidote from the corona. The antidote forms a double-stranded hybrid with TBA, thus restoring thrombin activity and blood coagulation. Fluorescently labeled TBA has a sequence of 5’ GGTTGGTGTGGTTGG-TMR 3’. The fluorescently labeled antidote has the complementary sequence 5’ CCAACCACACCAACC-FAM 3’. Clotting time (*t_plasma_*) for a thrombin test using 10 nM thrombin measured by a coagulometer with b) TBA, for c) 500 nM TBA + varying antidote from [anti]  =  0 to 1000 nM (anti/TBA  =  0 to 2.0).

Thrombin inhibitors are of great interest as candidates for anticoagulants because thrombin, which cleaves fibrinogen into fibrin to form the clot, is at the apex of the clotting cascade [11], [12]. We used ssDNA thrombin binding aptamers (TBA) to inhibit thrombin and consequently coagulation. We then used complementary DNA as an antidote because it can reverse TBA’s effect by base-pairing with it (Figure 1a). Selective excitation of two different NRs to release TBA and its antidote enables the pair to act as an on/off switch for coagulation.

## Results and Discussion

TBA has been identified by SELEX to bind and inhibit thrombin [13]. TBA folds into a double G-quartet and binds to exosite I, so it inhibits thrombin activity by preventing fibrinogen binding [14]–[16]. Thrombin inhibition results in an increase in blood clotting time, *t_plasma_*. We verified TBA’s ability to inhibit clotting by measuring its effect on *t_plasma_* in a thrombin test [17]. Blood with no TBA was normalized as *t_plasma_*  = 1.0. Increasing TBA concentration increased *t_plasma_* (Figure 1b), indicating that TBA inhibited thrombin and consequently the blood coagulation cascade.

We verified that the antidote could reverse TBA’s effect. As antidote:TBA was increased from 0:1 to 2:1, *t_plasma_* started at 4.4 and decreased (Figure 1c). Thus, antidote could successfully inhibit TBA by forming a double strand with it and preventing it from folding into the G-quartet structure necessary for thrombin binding (Figure 1a) [17]. ΔG_binding_ (TBA-thrombin)  = −35.6 kJ/mol, where ΔG_binding_ (TBA-antidote)  = −67 kJ/mol [15], [18], so TBA’s affinity for antidote is stronger than for thrombin. Also, we have previously demonstrated that in solution, the antidote can displace thrombin from TBA and that the TBA-antidote hybrid remains bound for several hours in the presence of thrombin [16]. Blood clotting time was restored to its original value at 1:1 TBA:antidote, indicating excess antidote was not needed to reverse TBA’s function [19], [20].

**Figure 2:**
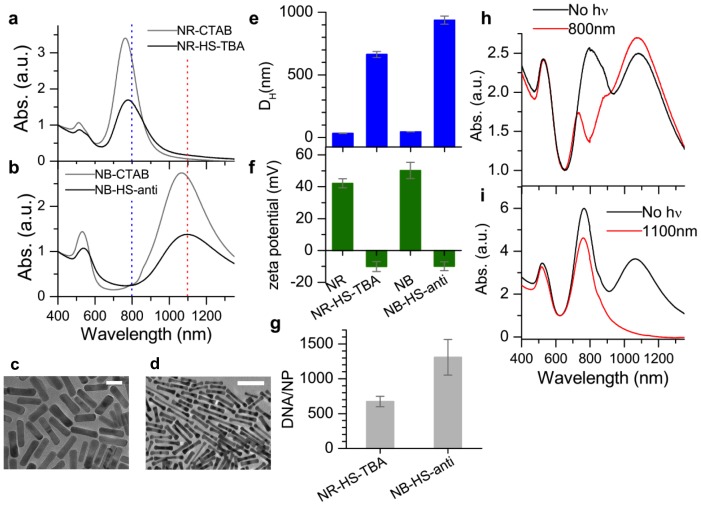
Gold nanoparticles synthesized and loaded for triggered release. Absorption spectra of a) NRs, NR-HSA-TBA coronas (LSPR max  = 777 nm), b) NB, NB-HSA-antidote (LSPR max  = 1093 nm). c) TEM image of NRs, scale bar  = 20 nm, d) TEM image of NBs, scale bar  = 100 nm, e) *D_H_* (DLS) of NRs, NR-HS-TBA, NBs, NB-HS-antidote, indicating that a corona contains multiple not a single NR or NB, but multiple ones. f) Zeta potential of NRs, NR-HS-TBA  = −9.8 mV, NBs, NB-HS-antidote  = −10.1 mV g) Quantified DNA payloads of NR-HS-TBA (674±74 TBA/NR), NB-HS-antidote (1307±255 antidote/NB). h) mixture of NR-CTAB + NB-CTAB before (black) and after (red) 800 nm irradiation. i) NR-CTAB + NB-CTAB before (black) and after (red) 1100 nm irradiation.

Cetyltrimethylammonium bromide (CTAB)-coated gold particles that absorbed at two distinct wavelengths were synthesized [21]–[23]. Rod-shaped nanorods (NRs) with an aspect ratio (AR)  = 3.7 and *D_H_* = 45 nm had a longitudinal surface plasmon resonance (LSPR) at 763 nm (Figure 2a, c, e). Bone-shaped “nanobones” (NBs) with AR = 4.8 and *D_H_* = 55 nm had a LSPR at 1065 nm (Figure 2b, d, e). Because NRs and NBs exhibited distinct absorption features, selective excitation by fs-pulsed lasers was possible. 800 nm irradiation of a NR-NB mixture resulted in melting of the NRs only, seen by a decrease of the LSPR at 800 nm (Figure 2h). Melting induced the NRs to change shape to spheres, which shifts and decreases their LSPR [24]. The LSPR at 1100 nm did not change, indicating that NBs were not melted because 800 nm coincides with a minimum in their absorption (Figure 2b, blue dotted line). Likewise, NRs do not absorb at 1100 nm (Figure 2a, red dotted line), so 1100 nm irradiation resulted in a decrease of the NB LSPR but did not affect the NR LSPR, indicating selective melting of the NBs (Figure 2i) [6]. Melting of the NRs and NBs separately was confirmed (Figure S1).

While DNA conjugation to gold NRs can be achieved by thiol-Au bonding [25], we used protein coronas because they exhibit high payload capacity while still enabling triggered release *via* laser excitation of the NR. Coronas of human serum (HS) were formed around the particles [26] and NR-coronas were loaded with TBA (NR-HS-TBA), NB-coronas with antidote (NB-HS-antidote). The NR-HS-TBA had *D_H_* = 662±25 nm, and NB-HS-antidote *D_H_* = 938±50 nm (Figure 2e, Figure S2), indicating that a corona contains multiple particles. However, NR and NB LSPRs were not significantly shifted (black, Figure 2a, b), so excitation at 800 and 1100 nm was still feasible. Zeta potentials for NR-HS-TBA and NB-HS-anti were negative because HS is negative (Figure 2f) [27]. DNA loading was 674±74 TBA/NR and 1307±255 antidote/NB (Figure 2g).

**Figure 3:**
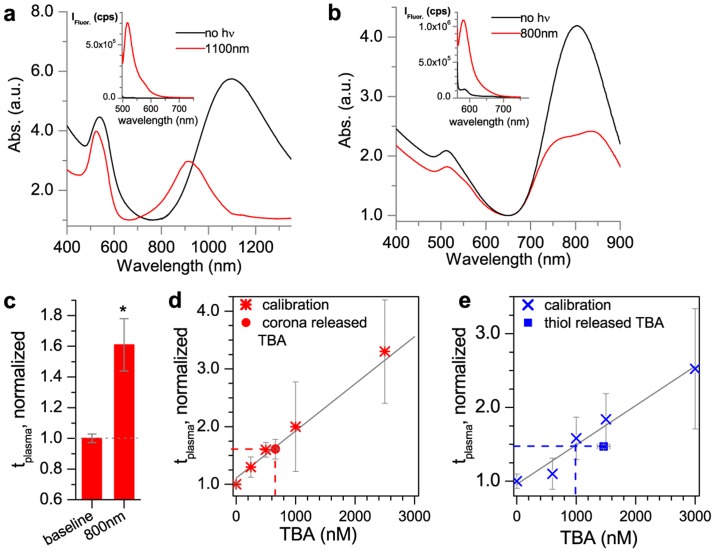
Release from NR- and NB-coronas and their comparison to covalently loaded NRs. a) Absorption spectrum of NB-HS-TBA before (black) and after (red) 1100 nm irradiation, where [NB-HS-anti]  = 0.3 nM, and released [anti]  = 129±5 nM (430±17 anti released/NB). Inset: fluorescence spectrum of released TBA before (black) and after (red) 1100 nm irradiation. b) Absorption spectrum of NR-HS-TBA before (black) and after (red) 800nm irradiation where [NR-HS-TBA]  = 2.9 nM, and released [TBA]  = 663±23 nM (223±8 DNA released/NR). Inset: fluorescence spectrum of released TBA before (black) and after (red) 800 nm irradiation, c) Effect of the released TBA in blood. Comparing normalized *t_plasma_* from released TBA from the coronas [NR-HS-TBA] = 2.9 nM (red), where released [TBA] = 663±23 nM in a clotting test. Supernatant of NR-HS-TBA with exposed to no irradiation and added to blood is defined as *t_plasma_*  = 1.0 (gray dotted line). A significant difference (p≤0.05) from baseline *t_plasma_* is indicated with a * (Table S1), d) *t_plasma_* (normalized) calibration curve of free TBA in a thrombin test (stars). Released TBA from NR-HS-TBA (red circle) and extrapolated equivalent concentration (red dashed line). e) *t_plasma_* (normalized) calibration curve of free thiolated TBA (blue X’s). Released thiolated TBA from NR-thiol-TBA (1460±108 nM, blue square) and extrapolated equivalent concentration (blue dashed line).

Laser irradiation could trigger DNA release. NB-HS-TBA were irradiated at 1100 nm and their LSPR decreased, confirming NB melting (Figure 3a). Released antidote quantified by fluorescence was [anti]  = 129±5 nM or 430±17 nM (Figure 3a, inset). NR-HS-TBA were irradiated at 800 nm and their LSPR decreased, confirming melting, and released [TBA]  = 663±23 nM or 223±8 released TBA/NR (Figure 3b, inset). Introducing released TBA to blood resulted in a *t_plasma_* increase to 1.61 (Figure 3c, red). Comparing the effect to free TBA, this change in *t_plasma_* would result from a free TBA concentration of [TBA]  = 609 nM (Figure 3d, red circle and dashed line). Thus, released TBA was ∼94% functional in coagulation, showing that there was minimal damage from the laser or steric hindrance by other released corona species. These experiments show that laser irradiation can release TBA from coronas on NRs, and released TBA is largely functional and can impact blood coagulation.

Using coronas for TBA loading has several advantages over covalent attachment. Compared to NRs conjugated to thiolated TBA (thiol-TBA), coronas had higher loadings and resulted in greater released DNA/NR. Analogous experiments performed with covalently attached thiol-TBA released less DNA/NR (released [thiol-TBA]  = 1460±108 nM, or 130±10 released thiol-TBA/NR), and released TBA was less functional (64%, blue circle, Figure 3e, and Figure S3). Furthermore, NR concentrations necessary to achieve this impact on *t_plasma_* were extremely high (11 nM), resulting in optically dense solutions that required long irradiation times (∼30 min). This in combination with the fact that the TBA was directly attached to the NR could be responsible for the lower activity due to laser damage [28]. Thus, using coronas for loading can reduce irradiation time and subsequently improve both yield and functionality of DNA. The coronas do have a broad size distribution (>35%), and it is expected that they have a distribution in the number of NRs or NBs per corona, and also amount of DNA per corona. This could potentially result in a distribution of the amount of DNA released per corona and also in the time it takes for the DNA to be released from the corona. However, the net benefits of the increased payload and the improved functionality of the released payload outweigh these disadvantages.

**Figure 4:**
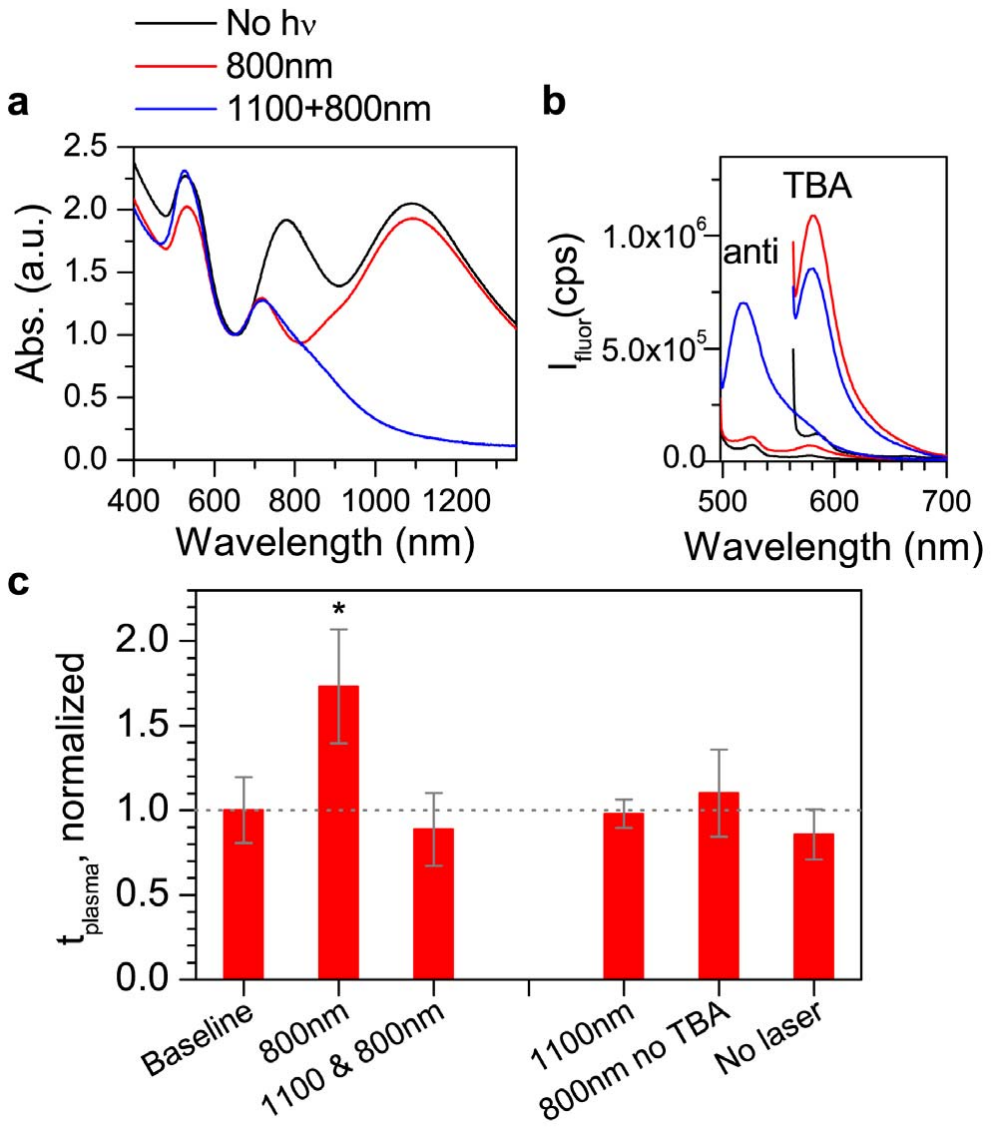
Selective melting of NRs and NBs to switch blood clotting off and on. NR-HS-TBA and NB-HS-antidote were mixed at a ratio so that the TBA:antidote was 1:1. a) NR-HS-TBA + NB-HS-anti mixture before (black) and after 800 nm irradiation (red), after 800 nm and then 1100 nm irradiation (blue) b) fluorescence of released supernatant before (black) and after 800 nm irradiation (red), and after 800 nm+1100 nm irradiation (blue). c) Normalized *t_plasma_* for samples before irradiation (defined as 1.0, and used to compare the statistical parameters of all samples) of mixture of NR-HS-TBA + NB-HS-antidote with excitation at 800 nm (*t_plasma_* increases to 1.73), and 800 nm+1100 nm (*t_plasma_* restored to 0.88), demonstrating restoration of clotting time. 1100 nm irradiation alone of the mixture NR-HS-TBA + NB-HS-anti, showing no significant increase in clotting time. Irradiation at 800 nm of NR-HS (without TBA) + NB-HS-anti showing no increase in clotting time. To test the effect of the presence of the nanoparticles, HS-NR-TBA+NB-HS-anti were not exposed to any irradiation in blood. Significant differences (p≤0.05) from baseline *t_plasma_* are indicated with an * (Table S1).

We selectively released TBA from the NRs and antidote from the NBs to inhibit and then restore blood clotting. First, a NR-HS-TBA + NB-HS-antidote mixture (black, Figure 4a) was exposed to 800 nm irradiation. The 800 nm LSPR decreased but the 1100 nm LSPR was unaffected, confirming selective melting (red, Figure 4a). TMR fluorescence in the supernatant due to released TBA increased while FAM fluorescence due to the antidote did not increase as much, illustrating that the NRs preferentially released payload (red, Figure 4b). Quantifying release showed that 800 nm irradiation released 107±4 nM TBA (252±10 released TBA/NR), but only 1.3±0.2 nM antidote. Released species introduced to blood resulted in an increase in *t_plasma_* to 1.73 (Figure 4c), indicating that released TBA inhibited thrombin and thus blood coagulation. Next, the NR-HS-TBA + NR-HS-antidote mixture was irradiated at 1100 nm. The 1100 nm LSPR decreased, confirming NB melting (blue, Figure 4a). FAM fluorescence increased, indicating that 152±18 nM antidote was released (692±82 released antidote/NB) (blue, Figure 4b), while 63 nM TBA was released. The effect on coagulation was *t_plasma_*  = 0.88, demonstrating that released antidote could reverse the effect of TBA and restore coagulation to its original *t_plasma_* (Figure 4c). 1100 nm irradiation alone on NR-HS-TBA + NB-HS-anti (Figure S4) did not significantly change *t_plasma_* (0.98, Figure 4c), showing that antidote release alone does not affect coagulation. To test whether HS release affects coagulation, NR-HS were prepared without TBA and mixed with NB-HS-anti. 800 nm irradiation resulted in no significant change in *t_plasma_* (1.1), confirming that the presence of TBA was necessary to affect coagulation. Finally, we verified that the presence of NR-HS-TBA + NB-HS-anti mixture with blood did not affect *t_plasma_* (0.86).

Timescales for melting NRs with an ultrafast laser pulse are on the order of ps [29], [30], and release and binding timescales are expected to be on the order of ns-ms. Overall, these timescales are more rapid than the coagulation experiment (∼20–200 s). The irradiation of the sample does take time (15min) due to the fact that the laser spot size is smaller than the sample. Timescales of minutes seem reasonable since the potential application of surgery has on operating timescale that is much longer. Also, the irradiation time could potentially be decreased by increasing the illumination area.

This work demonstrates that external laser excitation can selectively release a thrombin inhibitor and its antidote, allowing the NR/NB pair to act as an on/off switch for blood clotting. Furthermore, the use of protein coronas for loading and release of payloads from NRs opens up new possibilities for selective release applications, and it can be exploited for controlling blood clotting.

## Materials and Methods

### Synthesis and characterization of gold NRs

Gold nanorods (NRs) were synthesized by a single surfactant non-seed-mediated growth method in 20 mL batches [21], [23], [31] and gold nanobones (NBs) were synthesized in 50 ml batches by a double surfactant seed-mediated growth method [22]. TEM analysis showed that the NRs measured 34.6 ± 6.1 nm × 9.5 ± 2.2 nm, with an AR of 3.7 and the NBs measured 59.4 ± 13.7 nm × 12.5 ± 2.4 nm, with an AR of 4.8. All reagents were purchased from Sigma Aldrich, except from sodium chloride that was from Mallinckrodt. Excess reagents were removed by centrifuging the particles at 12000 rcf, 15 min and resuspended in MilliQ water. Afterwards, 1 nM NRs were further centrifuged at 12000 rcf for 15 min and resuspended in 5 mM CTAB for storage.

### Corona formation and loading

Coronas of human serum (HS) were formed around NRs and NBs and loaded with either TBA or antidote using a previously published combined loading approach [26]. Quantification of the payload was achieved by heating the sample for 30 min and 90°C and measuring the fluorescence spectroscopy of the supernatant. Briefly, a 20 μl pellet of 10 nM NRs was resuspended in 1 ml of a 5 mM phosphate buffer solution, with 1 μM ssDNA and 5% sterile filtered human serum, followed by an overnight incubation at 37°C, showing a loading of 674±74 DNA/NR and 1307±255 Anti/NB. Single stranded DNA (ssDNA) was purchased from Integrated DNA Technologies and fluorescently labeled for quantification with tetramethylrhodamine (TMR), or fluorescein (FAM). The sequences used were 5′ GGTTGGTGTGGTTGG-TMR 3′ (TBA) and 5′ AACCAACACAACCAA-FAM 3′ (anti). Human serum was purchased from Sigma Aldrich.

Corona formation was confirmed by UV-VIS, which was evidenced by a shift in the particle absorbance spectra, as well as DLS and zeta potential measurements, which showed an increase in the size of the particles and a change in the charge of the solution, which was due to the presence of the serum proteins.

### Selective release from NR-NB mixture

Laser irradiations were performed using pulsed femtosecond light. For the 800 nm irradiation, the 532 nm output of a Q-switched Nd:YLF laser (Empower, Spectra-Physics) was used to pump a Ti:Sapphire regenerative amplifier (Spitfire, Spectra-Physics), which amplifies the 82MHz output of a Ti:Sapphire oscillator, producing 50–475 μJ light centered at 800 nm, with a duration of 100 fs, a repetition rate of 1 kHz, and a spot size of 6 mm. The 1100 nm irradiation was achieved with a two-stage BBO/KNbO_3_ optical parametric amplifier, pumped with a 800nm pulse (Coherent Legend USX:<25 fs, 1 kHz), producing ***∼***13.6 μJ pulses, with a duration of ***∼***45 fs, centered at 1100 nm. The beam was focused to a spot size of ***∼***100–120 μm**.** In a typical experiment, 100 μl of the NR-NB mixture were irradiated in a 3×3 mm quartz cuvette for 20 min. After irradiation, samples were spun at 14000 rcf for 15 min, and the supernatants were collected to quantify the DNA released and for blood clotting tests. NP melting was confirmed by observing a decrease in their absorbance spectra.

### Blood clotting measurements

Plasma equivalent thrombin time was measured using a Hemochron Signature Elite (ITC). In a typical experiment, 14.4 μl of the sample were mixed with 1.8 μl of 100 nM thrombin and 1.8 μl of 1.37M NaCl, and incubated at 37°C for 10 min. After mixing with 27 μl of citrated whole blood, samples were loaded into APTT Citrate cuvettes to measure the blood clotting time. Blood was purchased from Research Blood Components (RBC) and used within 10 days. Functionality of the released TBA from coronas was calculated as a percentage between the measured increase of *t_plasma_* and the expected increase of *t_plasma_* from the same concentration of free TBA based on free TBA calibration curves.

## Supporting Information

Figure S1 **Melting the CTAB-NR and CTAB-NB: We separately tested melting of the NR-CTAB (a) and the NB-CTAB (b).** We could observe that the NR-CTAB melted after 800nm irradiation, and that the NB-CTAB could melt after irradiation at 1100nm, as evidenced by the decrease in their respective LSPR peaks.
(TIFF)

Figure S2 **Size distribution of coronas measured by DLS.** a) NRs (red) and NR-HS-TBA coronas (green), b) NBs (red) and NB-HS-antidote coronas (green).
(TIF)

Figure S3 **Melting and release of thiolated TBA from NRs.** a) Absorbance and b) fluorescence plots from the release of thiolated TBA bound covalently to the NRs. UV-VIS absorption shows a decrease of the SPR after the 800nm irradiation (left). Released thiolated TBA was quantified by fluorescence as 1460nM from 11.25nM NR-thiolated TBA (right), yielding 130.4 thiolated TBA released per NR.
(TIFF)

Figure S4 **Effect of irradiation at 1100 nm.** We irradiated the NR-HS-TBA NB-HS-anti mixture (before irradiation in black) at 1100nm. We observed melting of the NBs only (red line Figure S2), as observed by the decrease of their SPR at 1100nm, while the NRs were not melted. Inset shows the increase in fluorescence of the antidote, where 216nM antidote and 4nM TBA were released. The mixture had 0.37nM NB-HS-anti, and 0.40nM NR-HS-TBA.
(TIFF)

Table S1 **Statistics for blood clotting tests.** Two-tailed t-tests were performed using Origin 6.1. We report here the p-values of the samples that show significant differences. For each experiment, the baseline (no laser irradiation) was compared with the measured value of *t_plasma_*.
(PDF)

Text S1 **Supporting Information Synthesis: Method for loading thiolated TBA onto NRs by thiol chemistry.**
(PDF)

## References

[1] Wysowski DK, Nourjah P, Swartz L (2007) Bleeding complications with warfarin use – A prevalent adverse effect resulting in regulatory action. Arch Intern Med 167: 1414–1419. 17620536 10.1001/archinte.167.13.1414

[2] Shepherd G, Mohorn P, Yacoub K, May DW (2012) Adverse Drug Reaction Deaths Reported in United States Vital Statistics, 1999–2006. Ann Pharmacother 46: 169–175. 22253191 10.1345/aph.1P592

[3] Blossom DB, Kallen AJ, Patel PR, Elward A, Robinson L, et al.. (2008) Outbreak of Adverse Reactions Associated with Contaminated Heparin. N Engl J Med 359. 10.1056/NEJMoa0806450 PMC3810025 19052120

[4] Kishimoto TK, Viswanathan K, Ganguly T, Elankumaran S, Smith S, et al.. (2008) Contaminated heparin associated with adverse clinical events and activation of the contact system. N Engl J Med 358. 10.1056/NEJMoa0803200 PMC3778681 18434646

[5] Guerrini M, Beccati D, Shriver Z, Naggi A, Viswanathan K, et al.. (2008) Oversulfated chondroitin sulfate is a contaminant in heparin associated with adverse clinical events. Nat Biotechnol 26. 10.1038/nbt1407 PMC3491566 18437154

[6] Wijaya A, Schaffer SB, Pallares IG, Hamad-Schifferli K (2008) Selective Release of Multiple DNA Oligonucleotides from Gold Nanorods. ACS Nano 3: 80–86. 10.1021/nn800702n 19206252

[7] Huschka R, Zuloaga J, Knight MW, Brown LV, Nordlander P, et al (2011) Light-Induced Release of DNA from Gold Nanoparticles: Nanoshells and Nanorods. J Am Chem Soc 133: 12247–12255. 21736347 10.1021/ja204578e PMC4108302

[8] Lee SE, Sasaki DY, Park Y, Xu R, Brennan JS, et al (2012) Photonic Gene Circuits by Optically Addressable siRNA-Au Nanoantennas. ACS Nano 6: 7770–7780. 22827439 10.1021/nn301744x PMC3458151

[9] Barhoumi A, Huschka R, Bardhan R, Knight MW, Halas NJ (2009) Light-induced release of DNA from plasmon-resonant nanoparticles: Towards light-controlled gene therapy. Chem Phys Lett 482: 171–179.

[10] Yamashita S, Fukushima H, Akiyama Y, Niidome Y, Mori T, et al (2011) Controlled-release system of single-stranded DNA triggered by the photothermal effect of gold nanorods and its in vivo application. Bioorg Med Chem 19: 2130–2135. 21421321 10.1016/j.bmc.2011.02.042

[11] Kim Y, Phillips JA, Liu H, Kang H, Tan W (2009) Using photons to manipulate enzyme inhibition by an azobenzene-modified nucleic acid probe. Proc Natl Acad Sci U S A106: 6489–6494. 10.1073/pnas.0812402106 PMC2672545 19359478

[12] Lefkovits J, Topol EJ (1994) Direct thrombin inhibitors in cardiovascular medicine. Circulation 90: 1522–1536. 8087958 10.1161/01.cir.90.3.1522

[13] Bock LC, Griffin LC, Latham JA, Vermaas EH, Toole JJ (1992) Selection of single-stranded DNA molecules that bind and inhibit human thrombin. Nature 355: 564–566. 1741036 10.1038/355564a0

[14] Padmanabhan K, Padmanabhan KP, Ferrara JD, Sadler JE, Tulinsky A (1993) The structure of alpha-thrombin inhibited by a 15-mer single-stranded-DNA aptamer. J Biol Chem 268: 17651–17654. 8102368 10.2210/pdb1hut/pdb

[15] Pagano B, Martino L, Randazzo A, Giancola C (2008) Stability and binding properties of a modified thrombin binding aptamer. Biophys J 94: 562–569. 17890401 10.1529/biophysj.107.117382 PMC2157226

[16] de Puig H, Federici S, Baxamusa SH, Bergese P, Hamad-Schifferli K (2011) Quantifying the Nanomachinery of the Nanoparticle-Biomolecule Interface. Small 7: 2477–2484. 21692181 10.1002/smll.201100530

[17] Müller J, Freitag D, Mayer G, Pötzsch B (2008) Anticoagulant characteristics of HD1-22, a bivalent aptamer that specifically inhibits thrombin and prothrombinase. J Thromb Haemostasis 6: 2105–2112. 18826387 10.1111/j.1538-7836.2008.03162.x

[18] Markham NR, Zuker M (2005) DINAMelt web server for nucleic acid melting prediction. Nucleic Acids Res 33: W577–W581. 15980540 10.1093/nar/gki591 PMC1160267

[19] Rusconi CP, Roberts JD, Pitoc GA, Nimjee SM, White RR, et al (2004) Antidote-mediated control of an anticoagulant aptamer in vivo. Nat Biotechnol 22: 1423–1428. 15502817 10.1038/nbt1023

[20] Schwienhorst A (2006) Direct thrombin inhibitors–a survey of recent developments. Cell Mol Life Sci 63: 2773–2791. 17103113 10.1007/s00018-006-6219-z PMC11135997

[21] Sau TK, Murphy CJ (2004) Seeded High Yield Synthesis of Short Au Nanorods in Aqueous Solution. Langmuir 20: 6414–6420. 15248731 10.1021/la049463z

[22] Gou L, Murphy CJ (2005) Fine-Tuning the Shape of Gold Nanorods. Chem Mater: 3668–3672.

[23] Jana NR (2005) Gram-scale synthesis of soluble, near-monodisperse gold nanorods and other anisotropic nanoparticles. Small 1: 875–882. 17193542 10.1002/smll.200500014

[24] Link S, Burda C, Nikoobakht B, El-Sayed MA (2000) Laser-induced shape changes of colloidal gold nanorods using femtosecond and nanosecond laser pulses. J Phys Chem B 104: 6152–6163.

[25] Wijaya A, Hamad-Schifferli K (2008) Ligand Customization and DNA Functionalization of Gold Nanorods via Round-Trip Phase Transfer Ligand Exchange. Langmuir 24: 9966–9969. 18717601 10.1021/la8019205

[26] Kah JCY, Chen J, Zubieta A, Hamad-Schifferli K (2012) Exploiting the protein corona around gold nanorods for loading and triggered release. ACS Nano 6: 6730–6740. 22804333 10.1021/nn301389c

[27] Casals E, Pfaller T, Duschl A, Oostingh GJ, Puntes V (2010) Time Evolution of the Nanoparticle Protein Corona. ACS Nano 4: 3623–3632. 20553005 10.1021/nn901372t

[28] Jain PK, Qian W, El-Sayed MA (2006) Ultrafast Cooling of Photoexcited Electrons in Gold Nanoparticle−Thiolated DNA Conjugates Involves the Dissociation of the Gold−Thiol Bond. J Am Chem Soc 128: 2426–2433. 16478198 10.1021/ja056769z

[29] Huttmann G, Radt B, Serbin J, Birngruber R (2003) Inactivation of proteins by irradiation of gold nanoparticles with nano- and picosecond laser pulses. Proc SPIE 2003: 88–95.

[30] Link S, El-Sayed MA (1999) Spectral properties and relaxation dynamics of surface plasmon electronic oscillations in gold and silver nanodots and nanorods. J Phys Chem B 103: 8410–8426.

[31] Nikoobakht B, El-Sayed MA (2003) Preparation and growth mechanism of gold nanorods (NRs) using seed-mediated growth method. Chem Mater 15: 1957–1962.

