# Supplementary figures and images for: Selective Light-Triggered Release of DNA from Gold Nanorods Switches Blood Clotting On and Off
Source: PLoS One. 2013 Jul 24;8(7):e68511. doi: 10.1371/journal.pone.0068511 (PMC3722233; doi:10.1371/journal.pone.0068511)

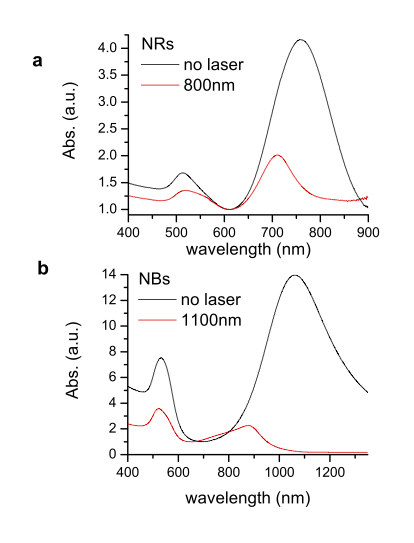

Supplement: Figure S1 — Melting the CTAB-NR and CTAB-NB: We separately tested melting of the NR-CTAB (a) and the NB-CTAB (b). We could observe that the NR-CTAB melted after 800nm irradiation, and that the NB-CTAB could melt after irradiation at 1100nm, as evidenced by the decrease in their respective LSPR peaks. (TIFF) [file pone.0068511.s001.tiff]

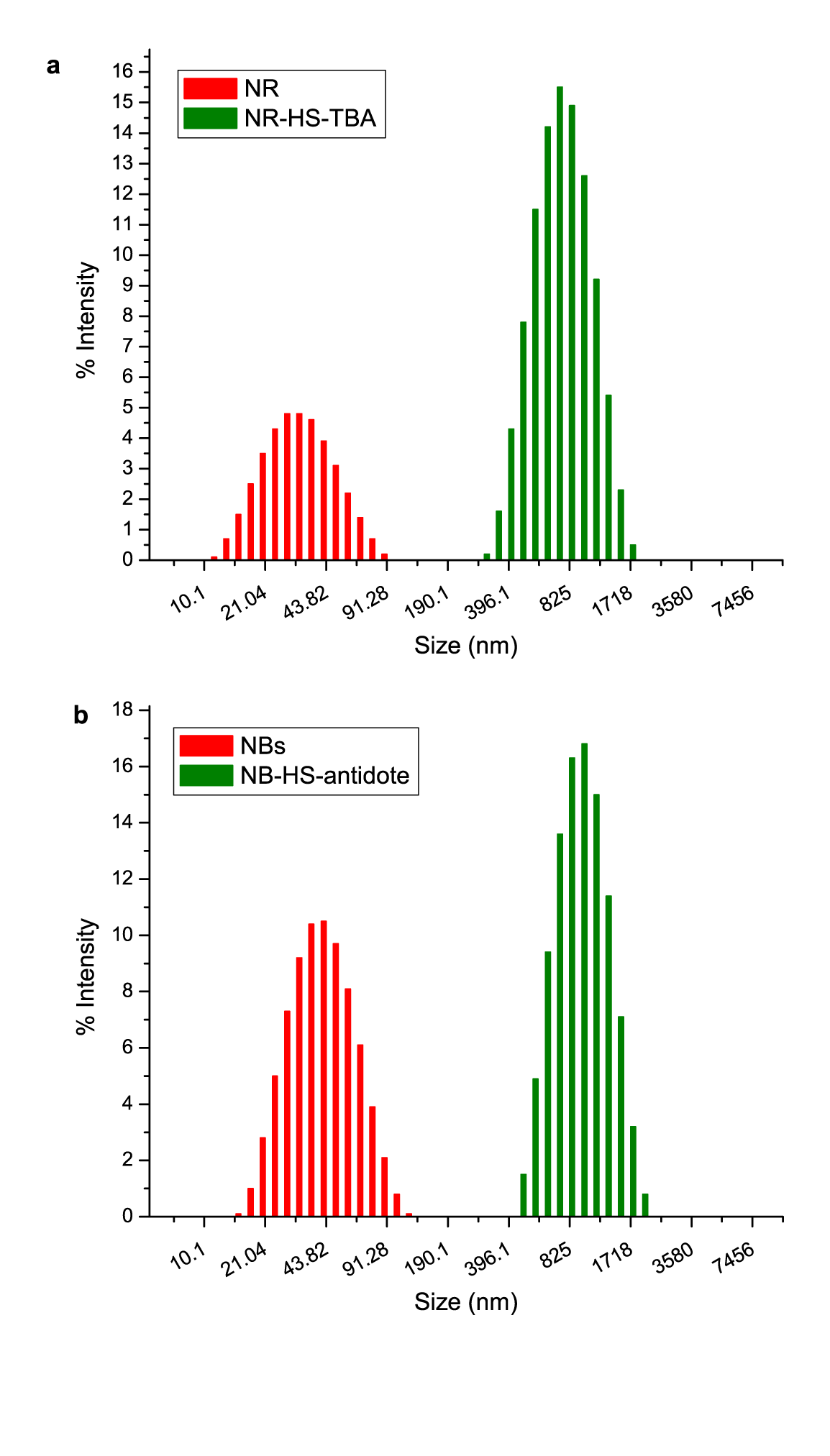

Supplement: Figure S2 — Size distribution of coronas measured by DLS. a) NRs (red) and NR-HS-TBA coronas (green), b) NBs (red) and NB-HS-antidote coronas (green). (TIF) [file pone.0068511.s002.tif]

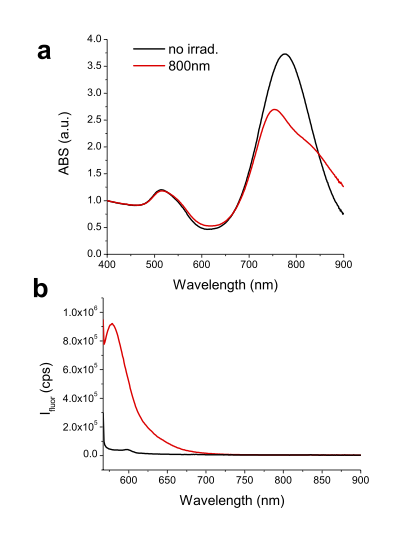

Supplement: Figure S3 — Melting and release of thiolated TBA from NRs. a) Absorbance and b) fluorescence plots from the release of thiolated TBA bound covalently to the NRs. UV-VIS absorption shows a decrease of the SPR after the 800nm irradiation (left). Released thiolated TBA was quantified by fluorescence as 1460nM from 11.25nM NR-thiolated TBA (right), yielding 130.4 thiolated TBA released per NR. (TIFF) [file pone.0068511.s003.tiff]

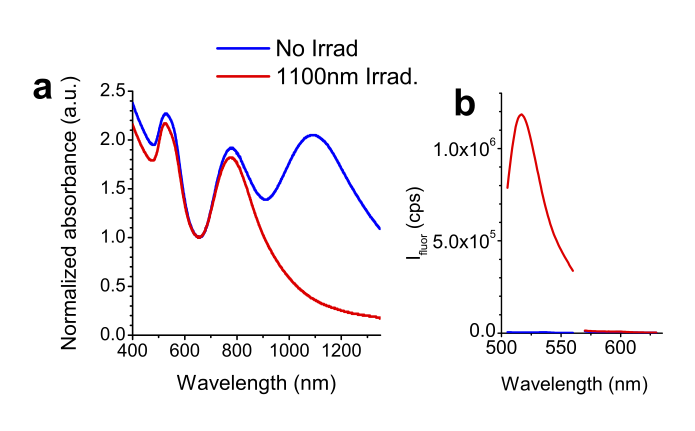

Supplement: Figure S4 — Effect of irradiation at 1100 nm. We irradiated the NR-HS-TBA NB-HS-anti mixture (before irradiation in black) at 1100nm. We observed melting of the NBs only (red line Figure S2), as observed by the decrease of their SPR at 1100nm, while the NRs were not melted. Inset shows the increase in fluorescence of the antidote, where 216nM antidote and 4nM TBA were released. The mixture had 0.37nM NB-HS-anti, and 0.40nM NR-HS-TBA. (TIFF) [file pone.0068511.s004.tiff]
