# Supplementary material for: Selective Light-Triggered Release of DNA from Gold Nanorods Switches Blood Clotting On and Off
Source: PLoS One. 2013 Jul 24;8(7):e68511. doi: 10.1371/journal.pone.0068511 (PMC3722233; doi:10.1371/journal.pone.0068511)
Supplement: Table S1 — Statistics for blood clotting tests. Two-tailed t-tests were performed using Origin 6.1. We report here the p-values of the samples that show significant differences. For each experiment, the baseline (no laser irradiation) was compared with the measured value of tplasma. (PDF) [file pone.0068511.s005.pdf]

Table S1: *Statistics for blood clotting tests.* Two-tailed t-tests were performed using Origin 6.1. We report here the p-values of the samples that show significant differences. For each experiment, the baseline (no laser irradiation) was compared with the measured value of t_{plasma}.

**Sample**	**Mean**	**s.d.**	**n**	**p value**
NR-Thiol-TBA<br>no irradiation	1.0	0.17	5	
NR-Thiol-TBA<br>800nm irradiation -*Figure S2b*	1.47	0.02	3	**3.16E-3**
NR-HS-TBA<br>no irradiation	1.0	0.16	6	
NR-HS-TBA<br>800nm irradiation -*Figure 3c*	1.61	0.17	5	**1.68E-4**
NR-HS-TBA+NB-HS-anti<br>no irradiation	1.0	0.2	10	
NR-HS-TBA+NB-HS-anti<br>800nm irradiation -*Figure 4c*	1.73	0.34	9	**2.02E-5**
