# Supplementary material for: Selective Light-Triggered Release of DNA from Gold Nanorods Switches Blood Clotting On and Off
Source: PLoS One. 2013 Jul 24;8(7):e68511. doi: 10.1371/journal.pone.0068511 (PMC3722233; doi:10.1371/journal.pone.0068511)
Supplement: Text S1 — Supporting Information Synthesis: Method for loading thiolated TBA onto NRs by thiol chemistry. (PDF) [file pone.0068511.s006.pdf]

Text S1

Methods for the thiolated TBA loading by thiol chemistry. The synthesized NRs were washed and concentrated by centrifugation (120 min at 7000 relative centrifugal force (RCF) for a 200 ml sample) to reduce their volume. Two more washes at 4000 RCF of 90 min each (the first one with 1mM CTAB, and the second with 10mM CTAB), were performed before storing the NRs in 1ml of 10mM CTAB.

The CTAB surfactant on the NR surface was exchanged with mercaptohexanoic acid (MHA) by a round-trip ligand exchange[1]; this method involves transferring the NR to an organic phase and then back to an aqueous phase with mercaptohexanoic acid (MHA) as the ligand.

In order to conjugate the NR-MHA with thiolated TBA, 6nM MHA-NR were incubated with thiolated-TBA in 10 mM phosphate buffer through a salt aging process[2]. The DNA oligonucleotides were purchased from Integrated DNA Technologies fluorescently labeled at the 3' ends with tetramethylrhodamine (TMR), and terminated with a 5' thiol (SH) for conjugation to the gold NRs, with sequence 5'-SH-GGTTGGTGTGGTTGG-TMR 3' (thiolated TBA).

Thiolated-TBA per NR was quantified by chemical displacement[3] with 1mM mercaptohexanol (MCH). Free DNA was quantified via fluorescence spectroscopy. Thiolated TBA coverage was 303 ± 61 thiolated TBA/NR, which is much higher than monolayer coverage. This high coverage is achieved due to the formation of TBA intramolecular G-quadruplexes.

References

1. Wijaya, A.; Hamad-Schifferli, K. *Langmuir* **2008,** 24, (18), 9966-9969.
2. Zu, Y.; Gao, Z. *Anal Chem* **2009,** 81, (20), 8523-8.
3. Park, S.; Brown, K. A.; Hamad-Schifferli, K. *Nano Letters* **2004,** 4, (10), 1925-1929.
